# Mechanosensitive calcium ion channels Piezo1: A therapeutic target in liver disease

**DOI:** 10.1016/j.omtn.2025.102695

**Published:** 2025-08-23

**Authors:** Jiao Liu, Xueying Wang, Fang He, Xiaoxu Chen, Xuejie Yi

**Affiliations:** 1 College of Exercise and Health, Shenyang Sport University, No. 36 Qiangsong East Road, Sujiatun District, Shenyang 110102, China; 2 School of Mechanical Engineering and Automation, Northeastern University, No. 11, Lane 3, Wenhua Road, Heping District, Shenyang 110819, China; 3 Exercise and Health Research Center/Department of Kinesiology, Shenyang Sport University, No.36 Qiangsong East Road, Sujiatun District, Shenyang 110115, Liaoning Province, China

**Keywords:** MT: Delivery Strategies, hepatic, signaling pathways, Piezo1, mechanism, fibrosis, HCC

## Abstract

The liver is vital in the metabolism of proteins, cholesterol, and carbohydrates, as well as energy production. Liver diseases often develop insidiously, making early diagnosis difficult, and rapid disease progression is directly associated with increased mortality risk. Ion channels, transmembrane proteins involved in various cellular functions, including development, migration, and programmed cell death, are associated with numerous diseases; however, the response of the liver to mechanical load and the underlying molecular mechanisms remain unclear. Piezo1, a mechanosensitive calcium ion (Ca^2+^ channel, is expressed in hepatic cholangiocytes, fibroblasts, stellate cells, Kupffer cells, and sinusoidal endothelial cells. Piezo1 has demonstrated potential relevance in liver pathophysiology; however, data on its therapeutic feasibility remain to be validated through systematic studies. This review aims to summarize Piezo1 expression and function; discuss its roles in fibrosis, hepatocellular carcinoma, iron metabolism disorders, and cholestasis; and distinguish established findings from hypotheses and highlight key gaps. Finally, considerations for the preclinical evaluation of Piezo1 modulators (e.g., *in vitro* assays, animal models, delivery, and safety) are outlined to guide translational research, rather than asserting definitive therapeutic efficacy at this stage.

## Graphical abstract

**Figure:**
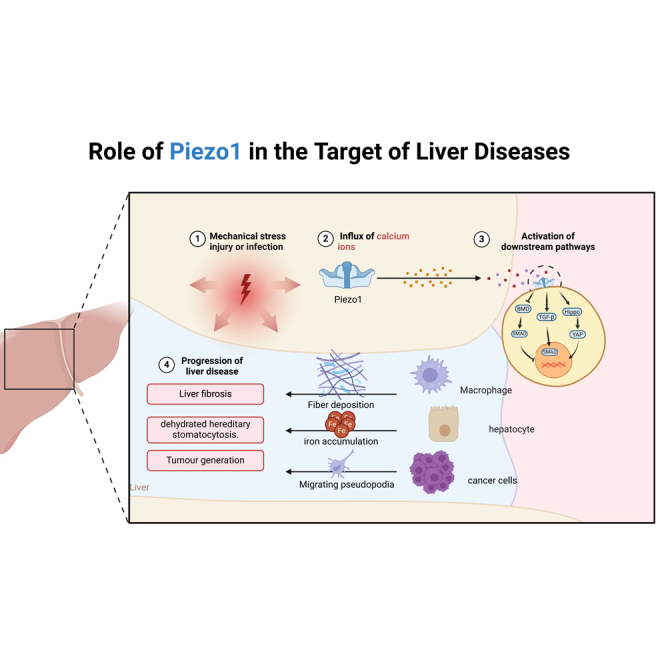

## Teaser

Piezo1, a mechanosensitive Ca^2+^ channel, plays pivotal roles in liver diseases by regulating fibrosis, hepatocellular carcinoma, and iron metabolism. This review summarizes its expression, functions, and therapeutic potential, highlighting key mechanisms and gaps in current research.

## Introduction

Liver disease remains one of the leading causes of morbidity and mortality worldwide, accounting for over 2 million deaths annually, primarily due to cirrhosis, viral hepatitis, and liver cancer, and representing approximately 4% of all global deaths (1 in 25).^1^ The global burden of liver diseases is expected to increase due to health-regulating factors, such as increased life expectancy, prolonged physical inactivity, and overnutrition, carrying significant implications for global health.^1^^,^^2^ Despite the high economic and human costs associated with liver diseases, treatment options remain scarce. Recent advances in liver phenomics have highlighted the molecular heterogeneity and complex pathophysiology underlying liver diseases, emphasizing the need for targeted and precise therapeutic strategies.^3^ Therefore, the development of specific medications for treating liver conditions is crucial for effective clinical management. Ion-channel-mediated calcium ion (Ca^2+^ signaling is a key regulator of liver physiology and pathology. Indeed, emerging evidence has highlighted the significance of ion channels in liver physiology and disease progression. Several recent studies have elucidated the roles of various ion channels in hepatic cellular functions and their involvement in pathological processes, including mechanotransduction and signal transduction pathways, which are essential to liver homeostasis and disease.^4^ In particular, the mechanosensitive channel Piezo1 has provided profound insights into the mechanotransduction in liver diseases and therapeutic targeting. Building on the crucial importance of Ca^2+^ signaling, recent research has uncovered the mechanosensitive channel Piezo1. Ion channels that mediate Ca^2+^ influx have become a focal point in liver disease research, owing to their extensive biological effects and significant pathological implications. Since winning the 2021 Nobel Prize in Physiology or Medicine, Patapoutian et al.’s discovery of the mechanosensitive cation channel Piezo1, made in 2010, has attracted considerable attention. Piezo1 is highly expressed in most cells, tissues, and organs of the human body and involved in sensing tension,^5^ immune responses,^6^ and cell proliferation.^7^ Currently, ectopic alterations in Piezo1 protein expression or function are being actively explored in diseases affecting the lung,^8^ kidney,^9^ heart,^10^ and gastrointestinal^11^ systems. Knockdown of Piezo expression at the genetic level has been shown to inhibit afferent action potentials and alleviate visceral hypersensitivity,^12^^,^^13^ suggesting Piezo1 as a potential therapeutic target. Recent studies have also demonstrated the importance of Piezo1 in the liver, particularly, liver diseases. Indeed, Luo et al.^14^ have reported that Piezo1 deletion in mouse models significantly reduces liver fibrosis, indicating its role in suppressing inflammatory responses and protecting liver function. Additionally, Wang et al.^15^ showed that Piezo1 enhanced the antifibrotic capacity of macrophages by promoting cytokine secretion, while Lichtenstein et al.^16^ indicated that Piezo1 could regulate cholesterol metabolism, affect lipid balance in the liver, and reduce the risk of liver disease. Despite the abundance of these data, systematic exploration of Piezo1’s precise regulatory mechanisms and roles in liver physiology and pathology remains lacking. Therefore, the objective of this review article is to provide a comprehensive narrative review of recent advances in research on the ion channel Piezo1 in liver diseases, aiming to offer insights for targeted therapy and drug development.

In this review, we summarize Piezo1 expression and function in hepatocytes and non-parenchymal liver cells (hepatic stellate cells [HSCs], Kupffer cells [KCs], and liver sinusoidal endothelial cells [LSECs]); discuss its involvement in fibrosis, hepatocellular carcinoma, iron overload, and cholestasis, differentiating established *in vitro/in vivo* findings from speculative hypotheses and highlighting key knowledge gaps; and outline considerations for preclinical evaluation of Piezo1 modulators in liver disease (e.g., dose-response assays, animal model validation, targeted delivery, and safety assessments) to guide translational research without claiming immediate clinical applicability.

## Piezo1 ion channel overview

Cells sense mechanical stimulation through membrane-expressed mechanosensitive receptors, a process known as mechanotransduction.^17^ Mechanical transduction is a fundamental aspect of signal transduction involving a network of mechanosensitive elements. The components include integrins and focal adhesions, which are essential for mechanosensing and mechanosignaling. For instance, integrins mediate the connection between the extracellular matrix and the cytoskeleton, allowing cells to sense mechanical forces and respond quickly.^18^ Focal adhesions serve as dynamic complexes that anchor cells to their surroundings and regulate cellular responses to mechanical stimuli.^19^^,^^20^ These elements orchestrate the cellular responses to mechanical forces, underscoring the multifaceted nature of mechanotransduction. Additionally, mechanosensitive ion channels are crucial components of mechanoreceptors. These channels, composed of membrane integrins, differ from traditional voltage-sensitive or ligand-gated ion channels and can detect changes in membrane tension induced by cell deformation, facilitating ion transport across the cell membrane and converting mechanical stimuli into electrical signals. Their unique ability to transduce mechanical and biochemical signals has garnered considerable attention in research in this area. As a key member of mechanosensitive ion channels, the discovery of Piezo1 has further advanced exploration in this field. The mechanosensitive ion channel is a gated ion channel composed of membrane integrins and differs from the traditional voltage-sensitive and ligand-gated ion channels. It can sense changes in membrane tension induced by cell deformation and other causes, resulting in the transmembrane transport of ions inside and outside the cell.

Furthermore, it can sense mechanical stimuli and convert them into electrical signals, owing to its unique ability to convert mechanical and biochemical signals through mechanosensitive ion channels. Among these, Piezo1 is an attractive and widely studied channel in life sciences and biomedical research due to its advantages, such as low energy consumption upon activation and high sensitivity to mechanical stimuli.^21^

Piezo1 and Piezo2 channels are present in most mammals and convert mechanical stimuli into biological signals.^22^ Piezo is assembled into a trimeric propeller-like ion channel with three blades: the central region of this channel is crucial for ion permeation, while the peripheral regions, comprising the three blades, are responsible for sensing mechanical force.^23^^,^^24^^,^^25^^,^^26^^,^^27^ A previous study demonstrated that Piezo1 underwent reversible deformation under mechanical stress.^5^ Yang et al.^28^ suggested that Piezo1 transitions from a curved to a flattened state in response to alterations in cell membrane tension. This shift opens the central pore, facilitating the conversion of mechanical signals into cation flows, as previously reported in structural and functional studies.^23^ These mechanosensitive channels respond to physical forces, such as shear stress, resulting in various plasma membrane tensions that activate them. Piezo1 is non-selective, allowing the permeation of univalent cations, such as K^+^ and Na^+^, along with divalent cations, such as Ca^2+^ and Mg^2+^, displaying a bias toward Ca^2+^.^22^^,^^29^^,^^30^ By mediating Ca^2+^ influx, they generate various intracellular Ca^2+^ signals critical in numerous biological processes. Piezo1 ion channels were initially discovered in mouse neuroblastoma cells,^29^ unlike mechanosensitive channels discovered in eukaryotes, such as transient receptor potential (TRP) channels, potassium channels, and DEG/ENaC channels, which are also thought to possess mechanosensitivity. This characteristic is more of an “additional attribute” for these channels.

While they can be activated by other physical or chemical stimuli, their mechanosensitivity is likely involved only in regulating or amplifying the activity of mechanosensitive channels of unknown molecular identity, playing an indirect role in mechanotransduction.^31^^,^^32^ Therefore, they cannot fully explain many mechanosensitivity-related physiological processes in mammals. Thus, Piezo ion channels are the real ion channel proteins proven to function as genuine mechanosensors.

**Figure 1:**
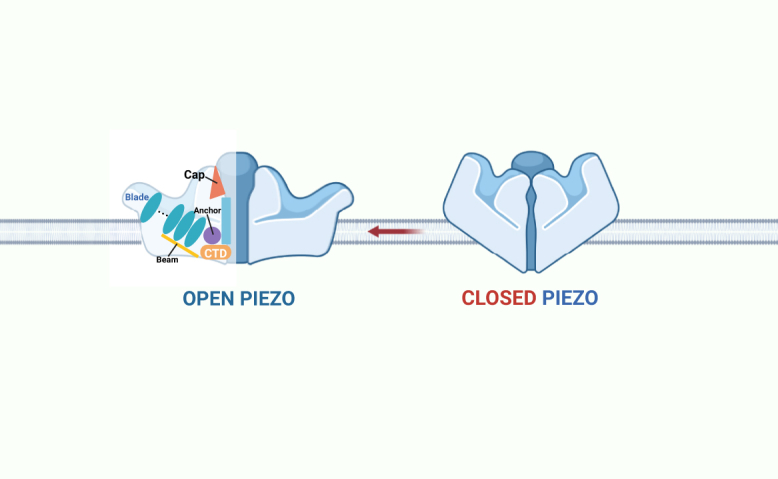
Piezo1 structure

Piezo1 channels on the cell membrane exhibit two forms, concave and flat,^28^ and comprise 38 transmembrane helical monomers,^24^ including an N-terminal blade involved in mechanosensation, a transduction unit that includes a beam and an anchor, and a C-terminal module for ion conduction.^17^ As mechanoreceptors on the cell membrane, Piezo1 channels sense mechanical stimuli, such as stretching and torsion, from the extracellular environment, converting these mechanical signals into electrical or chemical signals to elicit intracellular physiological responses. In mammals, including humans, the transmission of signals related to the perception of mechanical force is crucial for survival. In addition to mechanosensation, Piezo1 channels are vital for numerous physiological processes, including cell migration and differentiation,^33^ cardiovascular development,^34^ blood pressure regulation,^35^ and immune modulation.^36^ Given the physiological importance of Piezo1, gain-of-function (GOF) or loss-of-function mutations are associated with a range of pathological conditions due to resulting functional abnormalities.^17^ Piezo1 is involved in the regulation of multiple diseases, including bone metabolism disorders, fibrosis, cancer, and cardiovascular diseases. Figure 1 illustrates the cryo-EM-derived trimeric architecture of Piezo1.

Extensive research has focused on the three-dimensional (3D) structure and functional regulation of Piezo1 channels. Cryo-electron microscopy, X-ray crystallography, and single-molecule fluorescence microscopy, among other techniques, have revealed the structure and channel-gating mechanisms, offering profound insights into the functional regulation of Piezo1 channels. The mechanical microenvironment of the liver, shaped by interactions between cells and the surrounding extracellular matrix (ECM), as well as external mechanical forces, such as fluid shear stress (FSS), has a profound effect on liver function. As liver disease progresses, its mechanical properties, particularly tissue stiffness, increase, influencing the behavior of both parenchymal and non-parenchymal cells and triggering a series of electrochemical changes, including disruptions in calcium homeostasis and oxidative stress.^37^^,^^38^ Mechanotransduction in the liver, particularly through the Piezo1 channel protein, has emerged as a profound research focus in liver disease. Given Piezo1’s significant association with liver function and the current lack of comprehensive summaries in this field, further research is warranted to determine the role of Piezo1 channels in liver physiology and disease processes, as well as gain insights into therapeutic targets of Piezo1.

## Piezo1 expression and function in the liver

Recent studies have revealed that Piezo1 is expressed in the liver and plays a significant role in liver pathophysiology. The expression pattern of Piezo1 ion channels in the liver is complex, with expression sites including HSCs, LSECs, hepatocytes, and KCs. These cell types have different functions and localizations in the liver (Table 1). Several studies have demonstrated that Piezo1 expression in cells follows a multimodal distribution pattern, with varying degrees of expression in the biliary system,^39^ liver sinusoidal microvasculature, portal vein narrow areas,^34^^,^^43^ and liver parenchyma.

**Table 1:** Piezo1 expression and its role in liver-related diseases

Piezo1 channel	Expression	Related diseases	Main functions	References
Piezo1	hepatocytes	hepatic injury; hepatic fibrosis; cholestasis; hereditary xerocytosis	regulates the volume of hepatocytes; mediates liver injury induced by APAP; facilitates proliferation of hepatocytes and apoptosis induced by ROS; regulates the contraction cycle of bile ducts and bile secretion; controls iron metabolism through transcriptional repression of hepcidin and promotes liver iron overload	Gupta et al.^39^; Andolfo et al.^40^; Wang et al.^41^
	macrophage	hepatic	plays a key regulatory role in macrophage phagocytic activity and subsequent erythrocyte turnover; boosts the inflammatory response; and facilitates the revitalization and proliferation of HSCs	Luo et al.^14^; Ma et al.^42^
	liver sinusoidal endothelial cells	cholestasis; portal hypertension	controls force-induced ATP secretion in cholangiocytes, thereby regulating bile secretion; drives the recruitment of circulating blood cells; induces relaxation in the portal vein; and regulates first-pass metabolism, detoxification of blood components, and gluconeogenesis.	Hilscher et al.^43^; Endesh et al.^44^; Desplat et al.^45^

**Figure 2:**
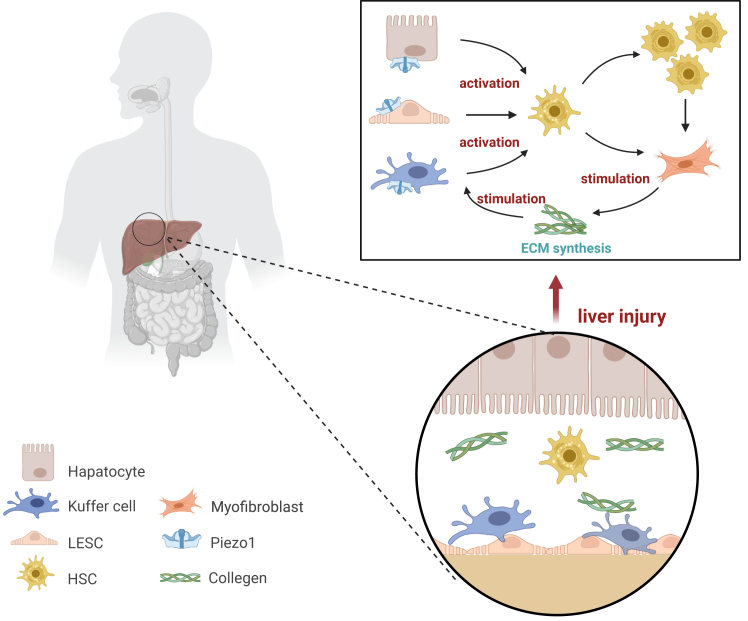
Crosstalk of cells in the liver during liver disease After chronic liver injury, hepatocyte homeostasis is disrupted, Piezo1 receptor is activated, and KCs, hepatocytes, and LSECs are activated. HSCs undergo phenotypic activation and proliferation and differentiate into fibroblasts, secreting a large amount of fibrotic ECM, which promotes ECM remodeling.

In particular, the expression of Piezo1 channels is the most prominent and highly specific in the liver microvasculature. Figure 2 illustrates a schematic overview of these cellular crosstalk events in liver disease.

### Parenchymal cells

#### Hepatocytes

Hepatocytes, the primary parenchymal cells of the liver, play a crucial role in carrying out the majority of the organ’s metabolic functions. They constitute a significant proportion of liver cells and are responsible for producing most of the proteins found in circulating plasma, including albumin, transport proteins, protease inhibitors, clotting factors, immune complexes, and inflammation regulators.^46^ The regulation of the intracellular Ca^2+^ concentration in hepatocytes is crucial for controlling numerous metabolic processes in the liver. Variations in cytoplasmic free Ca^2+^ levels within hepatocytes influence several key metabolic functions, including glucose metabolism, amino acid metabolism, drug metabolism, bile acid secretion, protein synthesis and secretion, lysosomal movement, vesicular dynamics, cellular proliferation, apoptosis, and necrosis. This intracellular signaling network plays a significant role in maintaining the physiological functions of hepatocytes and the liver as a whole.

Previous studies have confirmed the expression of Piezo1 in hepatocytes and AML12 cells.^25^^,^^26^^,^^27^^,^^39^^,^^40^^,^^47^ Acetaminophen (APAP)-induced swelling in AML12 cells upregulates Piezo1, accompanied by concomitant Ca^2+^ influx. Piezo1 knockdown exacerbates cell death, whereas Yoda1 activation attenuates APAP-induced apoptosis, necrosis, and mitochondrial oxidative stress.^41^ In an APAP-induced liver injury model, hepatocyte-specific overexpression of Piezo1 reduced serum ALT/AST levels by 38% and enhanced mitochondrial membrane potential retention by 55%. Conversely, Alb-Cre; Piezo1fl/fl mice (hepatocyte-specific Piezo1 knockout) exhibited a 2.1-fold increase in hepatocyte apoptosis compared with wild-type controls, demonstrating the protective role of Piezo1 in hepatocytes.

However, these observations derive from cell-based assays, and *in vivo* hepatocyte-specific validation is needed to confirm causality.^39^^,^^40^^,^^41^ Future studies should include dose-response evaluations of Piezo1 modulators (e.g., Yoda1 and GsMTx4) in animal models to establish therapeutic windows. In contrast, previous studies on APAP-induced hepatotoxicity have demonstrated that APAP disrupts cellular Ca^2+^ homeostasis, inducing oxidative stress and mitochondrial membrane permeability transition, further amplifying Ca^2+^ imbalance and intracellular Ca^2+^ increase, reducing mitochondrial potential, and halting ATP synthesis. Ultimately, ATP depletion leads to the death of hepatocytes.^48^^,^^49^^,^^50^^,^^51^^,^^52^ The cause of APAP-induced intracellular Ca^2+^ increase may primarily be attributed to the inhibition of Ca^2+^-Mg^2+^ ATPase, which accompanies (rather than causes) hepatocyte damage.^53^^,^^54^ Another possible reason is that, in APAP-induced liver injury, the Piezo1 activator Yoda1 enhances the protein levels of nuclear-factor-erythroid-2-related factor 2 (Nrf2), a crucial antioxidant regulator typically activated in response to oxidative stress. This mechanism helps combat oxidative damage and plays a pivotal role in protecting cells from APAP-induced acute liver injury (ALI).^55^ In APAP-induced ALI cells, Piezo1 activation mitigated APAP-induced mitochondrial membrane depolarization.^41^ Previous studies have suggested that the destruction of liver cell structure and function caused by infection, tissue stress, or injury alters the liver’s microenvironment. Furthermore, Piezo1 activation induced by infections inhibits the oxidative stress reaction, which may be indirectly caused by the potential side effects of the Piezo1 activator, Yoda1. Notably, hepatocyte-injury-released mechanical signals (e.g., ATP and HMGB1) can alter the stiffness of hepatic stellate cell microenvironment, activating their surface Piezo1; this transcellular signaling axis may constitute the critical bridge linking “hepatocyte injury to hepatic stellate cell activation” (see correlation analysis between sections hepatocytes and The regulation of liver tumors and liver cancer by Piezo1 ion channels).

### Non-parenchymal cells

The non-parenchymal areas of the liver are primarily composed of endothelial cells, which account for 19% of the total number of liver cells.^56^ Unlike other organs, the sinusoidal structure of liver endothelial cells lacks a basal layer, with no continuous barrier separating the epithelial cell surface from the plasma. Additionally, HSCs comprise 6% of liver cells, whereas KCs account for 15%.^46^ Non-parenchymal cells in the liver play a crucial role in liver immunity and are involved in regulating liver regeneration as important regulators of hepatocyte function.

#### Hepatic stellate cells

The activation of HSCs is a key factor in liver fibrosis. Piezo1 is essential for HSC activation and progression of liver fibrosis. Luo et al.^14^ reported that the deficiency of Piezo1 in macrophages limited liver fibrosis progression by inhibiting inflammatory responses. In chronic liver injury, mechanical stimulation or a prolonged inflammatory microenvironment leads to continuous and extensive activation of HSCs, facilitating their transformation into a myofibroblast-like phenotype with high proliferative capacity. These activated HSCs express high levels of α-SMA, fibrous collagen, elastin, and matrix proteins, enhancing the production and deposition of ECM and continuously secreting various inflammatory factors, exacerbating liver damage.^57^^,^^58^^,^^59^^,^^60^^,^^61^^,^^62^ HSCs are capable of moving and clustering in more rigid regions via mechanical signaling pathways, subsequently leading to further stiffening of the ECM.^63^ Currently, data on the precise manifestation of Piezo1 channels in HSCs are lacking.

Nevertheless, as critical nodes in liver fibrosis, HSCs alter the intracellular environment, creating conditions that promote cell activation and exhibiting behaviors similar to other stem cells. Within a matrix of specific stiffness, Piezo1 regulates whether the cellular matrix becomes more rigid or flexible. Additionally, induced Ca^2+^ transients can modulate gene expression and differentiation in stem cells.^64^ The differentiation of stem cells is controlled by mechanical stress through the stretch-activated ion channel Piezo1, which triggers cell proliferation and maintains tissue homeostasis by integrating various environmental cues.^64^^,^^65^^,^^66^^,^^67^ Piezo1 is expressed in the stem cells of various mammalian organs. Muscle stem cells (MuSCs) from Piezo1 knockout mice exhibit significantly reduced functional activity, displaying disease phenotypes similar to those of muscular dystrophy, indicating that Piezo1 plays a crucial role in muscle formation.^66^ The mechanical sensitivity of Piezo1 channels fosters distinctive stem cell differentiation *in vitro*. Piezo1 is a crucial mechanosensor for skeletal maturation and osteoblasts.^68^ Mechanical sensitivity of pressure receptors in mouse hair follicle stem cells depends on Piezo1.^69^ These findings indicate that stem cell fate can be influenced by mechanical stimulation via Piezo1 channels. Currently, there is no evidence to demonstrate that Piezo1 functions in HSCs; however, it has been hypothesized to regulate HSC activation and differentiation. Further research is needed, including profiling Piezo1 expression in quiescent versus activated HSCs and targeted manipulation *in vitro* or *in vivo*, to validate this hypothesis.

#### Kupffer cells

KCs, resident macrophages situated within the sinusoids, sense the tension and rigidity of the liver sinusoids, produce inflammatory mediators, and act as the primary defense mechanism within the liver immune system.^70^^,^^71^ KCs participate in phagocytosis, antigen presentation, as well as the generation of pro-inflammatory and pro-fibrotic cytokines, inducing inflammation, necrosis, regeneration, and fibrosis.^70^^,^^72^^,^^73^ Recent studies have emphasized the importance of extracellular mechanical stimulation on macrophage morphology, polarization, and function. Previous studies utilizing published transcriptomic datasets of mouse hepatic macrophages have analyzed the transcriptional levels of Piezo1 in these cells and identified a cluster of genes associated with Piezo1 expression in liver fibrosis tissue, providing promising research directions for investigating the role of Piezo1 in KCs.^15^ Recent studies demonstrate that constitutive or macrophage-specific expression of a GOF Piezo1 allele in mice disrupts the expression of the iron regulator hepcidin, leading to iron overload. Furthermore, these studies show that Piezo1 is a key regulator of macrophage phagocytic activity and subsequent erythrocyte turnover.^74^Although Piezo1-mediated mechanosensing has been demonstrated in other macrophages, its specific role in KCs remains unclear. Future work should clarify the changes in Piezo1 expression in diseases and test perturbations in relevant models (e.g., primary KC isolation and Piezo1 modulation in fibrosis models) to determine its functional significance. The inflammatory environment leads to abnormally high Piezo1 expression in macrophages, as well as an increase in the number of M1-type macrophages with pro-inflammatory properties and a significant increase in their infiltration. During this process, the interaction between macrophages and HSCs alters the immune system and ECM.^14^^,^^75^

Liver macrophages may detect ECM stiffening via mechanotransduction, subsequently differentiating into M1 or M2 phenotypes. This differentiation leads to the production of cytokines and chemokines as well as the regulation of HSCs through intercellular communication, ultimately influencing the progression and reversal of liver fibrosis. This suggests a significant interaction and interplay between mechanotransduction and biochemical signal transduction within the liver fibrosis process.^76^^,^^77^ The complexity of KC activation in disease underscores its ability to respond to dynamic changes in environmental stimuli. These findings suggest that Piezo1 channels play a significant role in liver immunity and fibrosis, highlighting the substantial research potential of liver macrophages.

#### Liver sinusoidal endothelial cells

The endothelial cells of the hepatic sinusoids, specialized endothelial cells within the hepatic microvascular unit, form the first barrier between liver tissue and blood circulation. The healthy liver contains HSCs positioned in the endothelial layer (LSECs) and epithelial layer (hepatocytes). In response to fibrotic signals, including inflammatory signals from hepatocytes and LSECs, HSCs change their phenotype into activated fibroblasts. Activated HSCs secrete fibrotic ECM, which, in the interstitial space, promotes fibrosis and impairs^78^^,^^79^^,^^80^ normal bidirectional metabolic exchange between portal blood flow and hepatocytes.^81^

Numerous studies have indicated that most endothelial cells express Piezo1 in the mRNA and protein forms.^33^^,^^34^^,^^82^^,^^83^^,^^84^^,^^85^^,^^86^ Sinusoidal endothelial cells in the liver experience mechanical stimulation from the bloodstream within the sinusoid, resulting in phenotypic alterations in HSCs and the release of ECM proteins.^87^ Piezo1 channels can detect the bloodstream and shear force and are linked to liver metabolic functions and the progression of fibrosis.^45^ Recent studies have identified Piezo1 channels in LSECs in both mice and humans,^34^^,^^43^^,^^45^^,^^83^ which affect vascular stability, including endothelial cell vasodilation, tension, and blood flow regulation.^44^ These channels also contribute to liver fibrosis by influencing the mechanosensing functions and biochemical pathways of HSCs.^14^^,^^43^^,^^73^ Additionally, higher expression levels of Piezo1 in the hepatic portal vein area could be linked to the biological effects stemming from portal hypertension in patients with cirrhosis. *In vitro* mechanical stretching activates Piezo1 in LSECs, promoting Notch1 targets (HE and HEY1) and neutrophil recruitment.^43^ However, *in vivo* validation in liver disease models is required to confirm their relevance (e.g., LSEC-specific Piezo1 perturbation in fibrosis or portal hypertension). Therefore, targeting mechanosensitive pathways to restore LSEC phenotypes is promising for treating and reversing chronic liver diseases, such as portal hypertension.

In summary, Piezo1 is primarily involved in regulating cell size and mitochondrial function in hepatocytes, thereby maintaining the normal physiological state of these cells. In contrast, in HSCs, it may influence the activation and differentiation of cells, which can contribute to hepatocyte development and participate in the process of liver fibrosis.

There may be a potential connection between the two. When hepatocytes are damaged, the release of signaling molecules may alter the microenvironmental mechanical forces in HSCs, which in turn affects the function of Piezo1 in HSCs, promoting its activation and the fibrosis process. Similarly, Piezo1-mediated immune responses in the KCs may interact with hepatocytes and HSCs to influence the development of liver disease, as illustrated in Figure 2. In-depth study of these mechanistic associations across cell types will contribute to a more comprehensive understanding of Piezo1’s roles in liver diseases and provide theoretical support for the development of more effective therapeutic strategies.

## Effect of Piezo1 ion channel on liver diseases

As the global living standards continue to improve, the prevalence of metabolic liver diseases, such as nonalcoholic fatty liver disease and alcohol-related liver disease, will increase. This will result in a higher incidence of end-stage liver diseases, such as liver failure, cirrhosis, and liver cancer.^57^ As a recently identified mechanosensitive cation channel, Piezo1 has been developed rapidly and exhibits great research potential for applications in liver disease. Piezo1 channels regulate the functions of hepatocytes and stellate cells, affecting physiological processes such as liver metabolism, immunity, and fibrosis. Furthermore, Piezo1 may be associated with various liver diseases, including hepatic iron overload, hepatitis, liver fibrosis, cirrhosis, and liver cancer.

**Figure 3:**
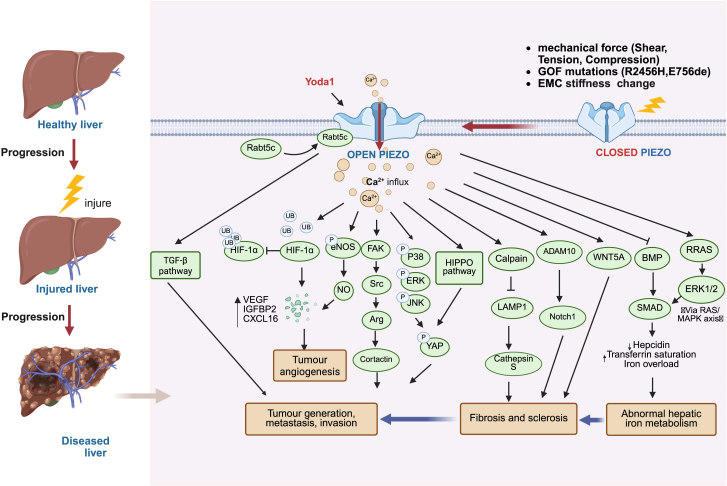
Mechanistic overview of Piezo1 involvement in liver disease progression, including regulation of fibrosis, hepatocellular carcinoma, and iron overload

Therefore, it is essential to gain a deeper understanding of the mechanisms of Piezo1 in the liver to effectively prevent and treat various liver diseases. Figure 3 summarizes the hypothesized signaling axes linking mechanical stress, Piezo1 activation, downstream effectors, and disease outcomes.

### Regulation of liver tumors and liver cancer by Piezo1 ion channels

There is increasing evidence suggesting Piezo1 as a potential drug target in liver cancer. Indeed, Li et al.^88^ showed that Piezo1 activation promotes hepatoma cell migration by 47% via TGF-β/Smad2/3 signaling, with an *in vivo* reduction in tumor volume of 38% upon Piezo1 knockdown (*p* < 0.01). Clinically, Fang et al.^89^ reported that high Piezo1 expression in multiple malignancies was correlated with hepatocellular carcinoma (HCC) (*p* < 0.001) and lower 5-year survival (*p* < 0.051), underscoring its prognostic value. Thus, targeting Piezo1 may offer promising therapeutic avenues for treating liver cancer. The expression patterns of Piezo1 channels are closely related to their roles in regulating cell migration *in situ*, cell morphology, and tumor growth. The tumor microenvironment plays a vital role in tumor progression. The ECM engages tumor and stromal cells, promoting the proliferation, movement, invasion, angiogenesis, and immune evasion of cancer cells. ECM and mechanical stimulation caused by ECM stiffening can activate membrane receptors and mechanoreceptors, thereby regulating the malignant phenotypes of neoplasms and basal plasma cells.^90^ Li et al. demonstrated that the initial factor of matrix stiffness enhances the activation of Piezo1 and the influx of Ca^2+^, which in turn inhibits the ubiquitination of HIF-1α, thereby facilitating the expression of pro-angiogenic factors VEGF, CXCL16, and IGFBP2, ultimately accelerating liver cancer angiogenesis.^91^ Angiogenesis is fundamental for tumor growth, invasion, and metastasis.^92^^,^^93^^,^^94^^,^^95^ Furthermore, in a high-stiffness Sprague Dawley (SD) rat orthotopic liver cancer model, a positive feedback regulatory loop involving stiff matrix/integrin β1/miR-625-5p/Piezo1 and COL1/stiffer matrix mediates the upregulation of Piezo1 expression by matrix stiffness.^91^ Similarly, Zhang et al.^96^ studied HCC cells grown on substrates with varying stiffness *in vitro*. Following treatment with Yoda1 (a Piezo1 activator) and GsMTx4 (a Piezo1 inhibitor), the authors observed a significant reversal in the migratory capacity and velocity of HCC cells, which were influenced by different levels of substrate stiffness. This suggests that Piezo1 activation and expression mediate matrix-stiffness-induced cell migration. Further research showed that inhibiting integrin β1 or Piezo1 significantly decreased the presence of invasive pseudopodia in HCC cells grown on substrates with high stiffness. Notably, the introduction of Yoda1 negated this reduction and, paradoxically, increased the presence of invasive pseudopodia. This finding underscores that activation of the integrin β1 or Piezo1/FAK/Src/Arg/cortactin pathway directly induces the formation of invasive pseudopodia in HCC cells. A rise in matrix rigidity may greatly enhance HCC cell migration through integrin β1 and Piezo1.^96^

The opening of Ca^2+^ ion channels allows Ca^2+^ to enter the cell through the plasma membrane. This process is crucial for regulating cellular activities associated with tumor development, including cell proliferation, migration, and apoptosis.^97^^,^^98^

Upon mechanical activation, Piezo1 mediates Ca^2+^ influx, which can promote FAK phosphorylation via Ca^2+^-dependent mechanisms (e.g., calmodulin/CaMKII-mediated regulation), as reported in other cell types.^99^^,^^100^ Although direct evidence in HCC cells is currently lacking, these studies support the plausibility of a Piezo1-Ca^2+^-FAK axis linking mechanical stimulus to downstream Src/Arg/cortactin-dependent cytoskeletal remodeling and invasive pseudopodia formation. Targeted experiments are required to validate the role of this axis in liver cancer. Liu et al. demonstrated that the HepG2 cell line expresses Piezo1 and that knocking out Piezo1 affects the proliferation, migration, and apoptosis of these cells. The activation of Piezo1 by Yoda1 promotes calcium influx, which in turn leads to the phosphorylation of JNK, p38, and ERK, thereby activating the MAPK pathway. By reducing Piezo1 expression and localizing Yes-associated protein (YAP) to the nucleus, a previous study has demonstrated that tumor growth in HepG2-derived liver cancer can be inhibited.^101^ This process also involves regulation of the Hippo/YAP pathway, which is essential for controlling organ size and cell growth.^102^ Future research should explore Piezo1 as a key factor in activating the Hippo-YAP/TAZ signaling pathway. As a mechanosensitive ion channel, Piezo1 senses mechanical stimuli and regulates intracellular calcium levels, which are closely linked to YAP/TAZ activity.^101^ Therefore, Piezo1 may indirectly modulate YAP/TAZ signaling by affecting cytoskeletal tension and calcium influx. In liver diseases, targeting Piezo1 could offer a promising therapeutic strategy by regulating YAP/TAZ signaling activity to mitigate liver fibrosis or inhibit liver cancer progression.

Vollmuth et al. indicated that the activation of Piezo1 advances Ca^2+^ influx in rat HSCs subjected to mechanical stimulation that mimics pathological hepatic blood flow and tissue stiffness, resulting in the release of WNT5A from HSCs and triggering apoptosis in hepatoma cells.^103^ Li et al.^88^ showed that the upregulation of Piezo1 promotes the *in situ* transfer of tumor cells, and overexpression of Piezo1 is closely associated with a poor prognosis in patients with liver cancer. The authors also indicated that inhibiting the activation of transforming growth factor β (TGF-β) signaling through Piezo1 reduces Rab5c recruitment, decreasing the proliferation and metastatic potential of liver cancer cells, diminishing their epithelial-mesenchymal transition capability, and making them more aggressive. Ye et al. also reported similar findings, showing that Piezo1 is significantly expressed in hepatoblastoma (HB) tissues and correlates with a poor prognosis in patients. Piezo1 can promote the proliferation of HepG2 and Huh6 cells and stimulate HIF-1α expression. Additionally, increased Piezo1 levels may promote HB cell movement and infiltration through the Piezo1/HIF-1α/VEGF axis.^104^ Piezo1 may act as a regulator of numerous signaling pathways and is involved in diverse forms of liver cancer through various pathways. The inactive ion channel Piezo1 can act on downstream effectors through the Hippo/YAP axis, thereby preventing cancer cell metastasis, inducing cell death, and inhibiting proliferation. This makes Piezo1 a potential target for the prognosis and treatment of intrahepatic cholangiocarcinoma.^105^ Piezo1 plays dual roles in cancer biology.

In liver fibrosis and HCC, Piezo1 activation mediates Ca^2+^ influx, leading to activation of pro-fibrotic and pro-tumorigenic pathways, such as TGF-β and Hippo/YAP signaling, thus promoting disease progression. Conversely, in other cancers, such as non-small cell lung carcinoma and gastric cancer, Piezo1 expression is often reduced and has been shown to inhibit cell migration and metastasis, potentially via integrin-mediated and Hippo-pathway-related mechanisms. These contrasting roles likely arise from differences in cellular context, tumor microenvironment, and downstream effectors, necessitating tissue-specific therapeutic approaches targeting Piezo1. Research indicates that Piezo1 expression is downregulated in non-small cell lung carcinoma cell lines, suggesting that Piezo1 acts as a tumor suppressor in lung cancer, inhibiting the migration and distant spread of lung cancer cells.^106^^,^^107^ Yang et al. have highlighted the significance of Piezo1 in gastric cancer. *In vitro*, Piezo1 has been shown to reduce the migratory ability of gastric cancer cells by downregulating integrin β subunits.^108^ The contradictions between these results may be attributed to the differences in the tissues examined. Piezo1’s pleiotropic roles in cancer reflect tissue-specific mechanotransduction programs: pro-fibrotic/pro-tumorigenic in liver diseases, yet tumor-suppressive in NSCLC and gastric cancer. In HCC, fibrotic stiffness (12–18 kPa) activates Piezo1-TGF-β/Smad2/3 axis, promoting MMP-9-driven metastasis. Conversely, in NSCLC, physiological lung compliance (4–8 kPa) is mediated by the Piezo1-Hippo/NF2 pathway, which enhances E-cadherin adhesion and suppresses invasion. These dichotomies arise from mechanosensitive threshold differences and downstream signaling plasticity, for example, Ca^2+^-dependent activation of TGF-β in liver versus Hippo in lung. Clinically, HCC tissues show Piezo1 overexpression (r = 0.68, with metastasis), whereas NSCLC tissues exhibit reduced Piezo1 expression (5-year survival of 62% in high vs. low expression). Such heterogeneity underscores the need for tumor-type-specific Piezo1-targeted strategies, such as TGF-β inhibitors in HCC and Hippo activators in NSCLC. Gastric cancer exhibits a distinct integrin-Piezo1-FAK axis, with rigidity-induced FAK phosphorylation enhancing migration. This distinction suggests that targeting Piezo1 requires different therapeutic strategies, depending on the cancer type.

### Regulation of liver fibrosis and cirrhosis by the Piezo1 ion channel

When the balance between the creation and breakdown of the hepatic matrix is disrupted, so is the equilibrium, leading to the deterioration of the liver tissue structure and the onset of diseases (such as fibrosis and cancer).^73^ The expression of Piezo1 in various mouse models has been extensively documented as being associated with the progression of fibrosis.^36^^,^^109^^,^^110^^,^^111^^,^^112^ Recent research has indicated that the knockout of macrophage Piezo1 in a mouse liver fibrosis model can inhibit the progression of liver fibrosis by reducing ECM deposition in the liver tissue, improving immune cell infiltration, inhibiting macrophage infiltration and polarization, and regulating T cell activation. Through *in vitro* experiments, Li et al. confirmed that the activation of macrophage Piezo1 can mediate M1 polarization and activation of HSCs, demonstrating that macrophages promote Ca^2+^ influx by activating Piezo1, regulating the transcription and activity of cathepsin S and the secretion of cathepsin S through the calpain/LAMP1 pathway, thereby regulating the progression of liver fibrosis.^14^ This study provides a preliminary explanation of the role of Piezo1 in the progression of liver fibrosis in macrophages.

Piezo1 overexpression activates signaling crosstalk in other cells, such as inflammatory responses, and stimulates HSCs, transforming their phenotype into activated fibroblasts, which increases liver fibrosis and stiffness. Liver stiffness is not merely a direct result of ECM accumulation and fibrosis; rather, it is a critical factor driving the progression of liver diseases, including fibrosis and cancer.^113^ As the ECM continues to accumulate, liver stiffness gradually increases, which in turn exacerbates conditions such as fibrosis and cancer. Additionally, liver stiffness influences resident liver cells through mechanotransduction, promoting a sustained fibrotic response and contributing to the formation of a pathological liver environment.^63^ Conversely, chronic liver disease increases ECM deposition, which in turn provides an impetus to resident liver cells, such as hepatocytes, portal vein fibroblasts, and HSCs. Therefore, investigating the signal transduction pathways in liver cells could significantly advance targeted therapy of fibrotic diseases. Vollmuth et al. indicated that Piezo1 contributes to the activation of the non-canonical WNT signaling pathway during liver fibrosis, significantly influencing the occurrence and progression of this disease.^103^ Notch1, a transmembrane receptor that binds to membrane-anchored ligands on adjacent cells, plays an important role in mechanically induced liver fibrosis. Hilscher et al. demonstrated that in LSECs, the interaction between mechanically responsive Piezo1 and integrins activates Piezo1 ion channels, which interact with the Notch1 receptor, inducing the activation of downstream transcription factors Hes1 and Hey1, ultimately upregulating the release of the CXCL1 chemokine and recruiting neutrophils to promote fibrosis.^43^ Caolo et al. proposed similar ideas concerning Piezo1 channels and Notch1 pathways. Their findings indicate that Piezo1 in hepatic endothelial cells is essential for regulating Notch1 expression, with activation likely driven by increased intracellular Ca^2+^ levels. Furthermore, the increase in ADAM10 enzyme activity leads to cleavage at Notch1’s S2 and S3 sites, thereby increasing the Notch1 intracellular domain and stimulating downstream signaling genes.^114^ Among the different liver cells, HSCs engage in the assembly of ECM proteins under the influence of thrombi and physical stress, thereby driving hepatic fibrosis.^73^ Although there is no clear evidence of the involvement of the Piezo1 signaling pathway in this process, validation in future experimental studies is highly feasible. In summary, as a Ca^2+^ influx channel that responds to mechanical stress, Piezo1 mediates a series of fibroblast movements upon activation, thereby forming a positive feedback loop that contributes to the progression of liver fibrosis and cirrhosis.^91^ The activation of Piezo1 ion channels regulates angiogenesis and fibroblast generation, promoting the progression of liver fibrosis into the common terminal pathway of chronic liver diseases. Nevertheless, the levels of Piezo1 expression in liver fibrosis and cirrhosis vary, necessitating further investigation to elucidate Piezo1’s downstream signaling pathways and its molecular mechanisms in the treatment of liver fibrosis.

### Regulation of liver iron overload by Piezo1 ion channel

Iron plays a crucial role in cellular biological functions, including the synthesis of hemoglobin for oxygen transport. Disruption of iron homeostasis is not only a contributing factor to various diseases—such as atherosclerosis, cardiomyopathy, renal ischemia-reperfusion injury, and neurodegenerative disorders^115^—but also a cause of chronic liver disease^116^ and HCC metastasis.^117^ Iron overload in the liver leads to hepatic fibrosis and cirrhosis, accelerating the progression of non-alcoholic fatty liver diseases.^118^ Most proteins involved in iron metabolism, including hepcidin, ferritin, and transferrin, are produced in the liver.^119^ Ferritin is a protein that sequesters iron, allowing cells to store it safely.^120^ Transferrin is characterized by its reversible iron-binding capacity, whereas hepcidin serves as the principal regulator of systemic iron metabolism. Hepatocytes transcribe and translate the gene encoding the peptide hormone hepcidin in response to elevated iron levels. Hepcidin maintains iron homeostasis by downregulating iron transporter activity, thereby inhibiting iron release from intestinal epithelial cells and macrophages into the bloodstream.^121^

Clinical data from patients with hereditary xerocytosis demonstrate that individuals with Piezo1 gene mutations exhibit significant iron metabolism dysregulation, with some patients presenting liver iron overload as a primary clinical feature, suggesting an initial link between Piezo1 and hepatic iron metabolism.^42^^,^^122^^,^^123^^,^^124^ Recent studies have corroborated this association. Using hepatocyte cell lines, Andolfo et al. investigated the expression of key iron metabolism mediators in an *in vitro* model. Expression of the R2456H and R2488Q GOF Piezo1 mutants enhanced Ca^2+^ influx more robustly than wild-type Piezo1. Their study established a relationship between altered Piezo1 channel function and impaired iron metabolism, indicating that GOF Piezo1 mutations can inhibit hepatic iron regulation by suppressing the bone morphogenetic protein (BMP)/SMAD pathway.^40^ However, limited evidence suggests that liver iron overload associated with GOF Piezo1 may be secondary to erythrocyte dehydration in hereditary xerocytosis.^42^ This assumption was challenged by Ma et al., who reported that aged mice with specific Piezo1 GOF mutations exhibited progressive hepatic iron accumulation compared to wild-type controls. Serum transferrin saturation and ferritin levels were elevated, and increased hepatic macrophage iron staining was observed. In homozygous Piezo1 mutant mice, iron deposition is more pronounced than in heterozygotes, indicating age-dependent hepatic iron overload.^74^ Similarly, in a cohort of African American patients over the age of 40 years carrying the E756del Piezo1 polymorphism, comparable elevations in serum ferritin, transferrin saturation, and hepcidin levels were observed. The proposed mechanism involves Piezo1-mediated regulation of macrophage phagocytic function and erythrocyte turnover, ultimately influencing *in vivo* hepcidin expression. Macrophages expressing E756del Piezo1 exhibited increased erythrocyte turnover, resulting in elevated erythroferrone levels in adult mice and reduced hepcidin expression.^125^ These findings suggest that hepcidin dysregulation due to GOF Piezo1 mutations is a central mechanism underlying age-related iron overload, offering profound insights into iron deposition in aging populations. Collectively, these data support the notion that Piezo1 inhibition alleviates iron overload by regulating iron storage and efflux, thereby improving hepatic iron metabolism and potentially delaying chronic liver disease progression. Recent evidence also revealed that Piezo1 GOF variants regulate hepatic iron overload through the RAS/MAPK axis, by promoting RRAS activation and ERK1/2 phosphorylation, which in turn inhibits BMP/SMAD-dependent hepcidin transcription. This crosstalk expands our understanding of the Piezo1-mediated hepcidin suppression beyond the Ca^2+^ influx alone.^126^

Although whole-body Piezo1 GOF mutations and mutant mouse models have demonstrated hepatic iron overload, the cell-specific role of Piezo1 within hepatocytes remains unexplored. To date, no studies have employed hepatocyte-specific Piezo1 deletion (e.g., Alb-Cre-driven knockout) to evaluate effects on hepcidin expression, serum iron parameters, or hepatic iron deposition. Future research should utilize hepatocyte-specific Piezo1 knockout models to assess hepcidin mRNA/protein expression, serum ferritin and transferrin saturation levels, and liver iron staining and compare these findings with macrophage-specific knockout models to delineate cell-autonomous versus non-cell-autonomous contributions to iron homeostasis.

### Regulation of other liver diseases by Piezo1 ion channel

The regulatory functions of the Piezo1 ion channel in hepatic fibrosis, cirrhosis, and hepatocellular carcinoma have been previously discussed; however, emerging research suggests that Piezo1 also plays a critical role in other liver diseases.

Multiple studies have explored Piezo1-mediated Ca^2+^ regulation to gain multi-scale insights into mechanosensing in liver physiology. Wang et al. observed upregulation of Piezo1 expression in hepatocytes of mice with APAP-induced acute liver injury (ALI). The proposed mechanism involves cell-swelling-induced membrane tension, which activates Piezo1 and alleviates APAP-induced cell death and mitochondrial oxidative stress by upregulating the Nrf2–Nqo1/Gsta1 pathway.^41^

Portal hypertension (PHTN), a common complication of chronic liver disease, is a leading cause of mortality and liver transplantation in patients with cirrhosis. Endesh et al. have demonstrated that activation of Piezo1 in portal venous endothelial cells upregulates nitric oxide synthase and enhances endothelium-dependent relaxation. Mechanical and osmotic stress amplified Piezo1-mediated vasorelaxation while concurrently suppressing TRPV4-induced vasoconstriction.^44^ These effects promote portal vein dilation and improve hepatic blood flow.

In a murine model of portal hypertension induced by congestive liver disease, Hilscher et al. administered the neutrophil elastase inhibitor sivelestat or performed bile duct ligation. The authors found that mechanical stretch activated Piezo1 signaling in LSECs, leading to the upregulation and release of the neutrophil chemokine CXCL1. This chemokine recruited neutrophils, which interacted with platelets to promote neutrophil extracellular trap (NET) formation and hepatic thrombosis. Inhibiting NET formation alleviates PHTN.^43^ In another portal hypertension model, Desplat et al. identified a Piezo1-Panx1 signaling axis in cholangiocytes.

Hypoosmotic stress triggers calcium influx and ATP secretion via Piezo1 activation, affecting ductal bile secretion. Piezo1 functioned as a mechanosensor, transducing membrane stretch into Panx1-mediated ATP release.^45^ The secreted ATP, in turn, acts in an autocrine or paracrine manner through P2X4R receptors to coordinate cholangiocyte responses and regulate bile formation and secretion.^127^^,^^128^ These findings suggest Piezo1 as a promising therapeutic target for the management of cholestatic liver disorders.

Further research remains warranted to elucidate the diverse roles of Piezo1 in liver pathophysiology. Given the limited literature on targeting Piezo1 for liver disease therapy, future investigations should focus on elucidating Piezo1 mechanisms across various liver disease subtypes, with a particular emphasis on the therapeutic potential of Piezo1 modulation.

## Conclusions

This review has highlighted current findings regarding Piezo1 expression and function in hepatic cell populations, mapped relevant signaling mechanisms, and underscored key knowledge gaps, including the need for cell-type-specific *in vivo* models and translational strategies. Accumulating evidence implicates Piezo1-mediated mechanotransduction as a central contributor to liver disease progression, from fibrosis to HCC. Future research should validate Piezo1-targeted modulators in appropriate models, uncover tissue-specific regulatory mechanisms of Piezo1 activity, and develop effective therapeutic delivery approaches to leverage Piezo1 as a possible clinical target in liver disease.

Piezo1, a recently identified mechanosensitive cation channel, plays a pivotal role in Ca^2+^ influx triggered by mechanical strain and intracellular environmental changes. Increased membrane tension from cellular traction activates Piezo1, initiating functional responses across diverse cell types and promoting the secretion of regulatory factors in response to mechanical stimuli.^129^ Owing to its broad tissue distribution and pleiotropic effects, Piezo1 is a compelling target for tissue-specific drug development. Therefore, Piezo1 agonists and inhibitors are being investigated as potential therapeutics for a wide spectrum of human disorders, including acquired and genetic diseases.

Although research on Piezo1 channel inhibitors is still in its early stages, notable progress has been made in the development of selective agents. These inhibitors can be broadly categorized based on their mechanisms of action^29^^,^^130^^,^^131^^,^^132^^,^^133^^,^^134^^,^^135^^,^^136^^,^^137^:

(1) Pore blockers, which directly occlude the ion conduction pore of the channel, including ruthenium red (RR)^32^^,^^133^ and gadolinium (Gd^3+^).^134^^,^^135^^,^^136^
(2) Lipid bilayer modulators, which alter channel activity via interactions with surrounding membrane lipids, such as GsMTx4^137^ and amyloid-β peptides.^138^
(3) Competitive antagonists, which block Piezo1 by competing with its synthetic activator Yoda1 at the binding site, including Dooku1,^139^^,^^140^^,^^141^ Tubeimoside I,^142^ and salvianolic acid B.^130^

Although preclinical results have been promising, Piezo1 inhibitors encounter significant hurdles that must be addressed prior to clinical application. Their efficacy varies depending on cell type and microenvironment,^131^ and off-target effects due to limited specificity remain a significant concern.^143^ Liver-specific targeting is particularly difficult, as systemic delivery poses a risk of adverse effects in non-hepatic tissues. Advances in structure-guided drug design, informed by high-resolution cryo-EM structures of Piezo1, may enhance the selectivity and potency of inhibitors. Additionally, liver-directed delivery platforms—such as nanoparticle carriers or ligand-conjugated prodrugs—are crucial for concentrating therapeutic agents within hepatic tissue while minimizing systemic exposure. The development of companion diagnostics to stratify patients based on Piezo1 expression or genetic variants could further improve therapeutic efficacy and safety. Together, these strategies underscore the therapeutic potential of Piezo1 modulation in liver disease and the considerable barriers that remain.

Current Piezo1 modulators, such as the peptide inhibitor GsMTx4, have been shown to block Piezo1-mediated currents *in vitro*.^144^ Conversely, small-molecule agonists, such as Yoda1, can activate Piezo1 but exhibit poor pharmacokinetic profiles and potential off-target effects.^131^ Structural insights from cryo-EM studies provide a foundation for rational drug design, aiming to optimize affinity and specificity.^25^ Liver-targeted delivery strategies—for instance, nanoparticle-based systems or ligand-directed conjugates—have shown efficacy for other ion channel modulators and could be adapted to Piezo1-targeted therapies.^145^ However, preclinical evaluation of Piezo1 modulators remains limited, and no compounds have yet advanced to registered clinical trials for liver disease indications.^131^ Biomarker-based patient stratification using Piezo1 expression levels or mutation profiles may enhance treatment outcomes, particularly since expression levels vary across tissues and disease states.^146^

Future research should prioritize high-throughput compound screening, structure-based lead optimization, and validation in organoid and animal models that accurately replicate human liver pathophysiology.^131^ Progress in this area will require close collaboration among structural biologists, medicinal chemists, pharmacologists, and clinical researchers to translate Piezo1-targeted therapies into clinical practice.

In summary, the mechanosensitive cation channel Piezo1 plays a critical role in hepatic pathophysiology, where mechanical-stimuli-induced alterations in Ca^2+^ homeostasis contribute to the progression of liver fibrosis, cirrhosis, and cancer. Piezo1 activity appears to influence multiple disease-related biological processes, including oxidative stress, inflammation, apoptosis, and cell proliferation. Thus, modulating Piezo1 expression or activity represents a promising therapeutic avenue for liver disease. Future investigations should delve deeper into the regulatory functions of Piezo1, with a particular focus on its role in liver biology, diagnostic utility, and therapeutic potential. Moreover, maximizing the clinical value of Piezo1-targeted drug development will require a deeper understanding of tissue-specific modulators of Piezo1 sensitivity.

## Acknowledgments

The author(s) declared that financial support was received for the research, authorship, and/or publication of this article. This paper was supported by the 10.13039/501100005047Liaoning Provincial Natural Science Foundation Program Upper Fund project 2024-MS-220 and Liaoning Provincial Department of Education Basic Research Project LJ212410176008. The author acknowledges BioRender for providing the drawing platform. We are grateful to Miss Li for their assistance with securing the figure copyright permissions. We would like to thank Editage (www.editage.cn) for English language editing.

## Author contributions

X.W., methodology, validation, and writing—original draft. F.H., methodology, validation, and writing—original. X.C., validation, methodology, and writing—original draft. X.Y., writing—review and editing and validation. J.L., conceptualization, funding acquisition, methodology, project administration, writing—review and editing, and supervision.

## Declaration of interests

The authors declare that the research was conducted in the absence of any commercial or financial relationships that could be construed as a potential conflict of interest.
